# Comment on: Emergence of the invasive malaria vector *Anopheles stephensi* in Khartoum State, Central Sudan

**DOI:** 10.1186/s13071-021-05080-y

**Published:** 2021-11-27

**Authors:** Jan Kolaczinski, Samira Al-Eryani, Emmanuel Chanda, Lucia Fernandez-Montoya

**Affiliations:** 1 World Health Organization, Headquarters, 1211 Geneva, Switzerland; 2 World Health Organization, Eastern Mediterranean Regional Office, Cairo, 11371 Egypt; 3 World Health Organization, Africa Regional Office, Brazzaville, Republic of Congo

**Keywords:** *Anopheles stephensi*, Sudan, Horn of Africa, Invasion, Response, Surveillance, Reporting, Global Vector Control Response

## Abstract

This letter comments on the article “Emergence of the invasive malaria vector *Anopheles stephensi* in Khartoum State, Central Sudan” published in *Parasites and Vectors* 2021, 14:511. Here we aim of provide a response to this paper in the broader context of the invasion and spread of *An. stephensi* in the Horn of Africa, and the required response to it. We agree with the authors that the arrival of this invasive vector in Khartoum State is of high public health concern. Equally concerning, however, we found that the detection of the vector by the authors in 2018 seemingly took 3 years to communicate to the Ministry of Health and World Health Organization (WHO), and was reliant on an academic journal. We consider that this short report sets a poor example of how public health threats should be reported. Suitable communication alternatives to alert public health authorities to such threats have been put in place by the WHO and its Member States, and are well known to at least some of the authors of the short report. We would like to encourage all readers not to follow the example of Ahmed et al. but instead act as responsible public health professionals by drawing on the established reporting mechanisms and escalate potential threats as soon as they are identified.

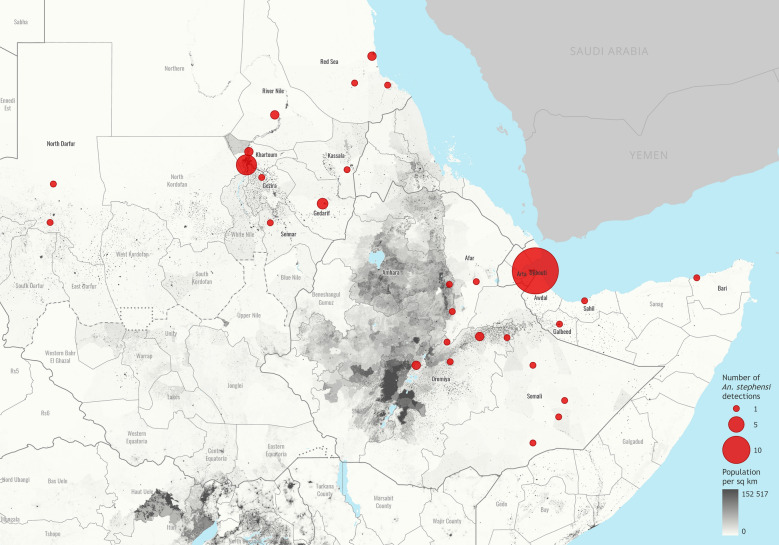

## Letter to the Editor

Recently, Ahmed and colleagues have published an alarming report on the detection of the invasive malaria vector *An. stephensi* in 2018 in the center of Khartoum, the capital city of Sudan with a population of over 5 million inhabitants. This adds yet another report to a growing list of localities from where the vector has been reported in the Horn of Africa (https://apps.who.int/malaria/maps/threats/), a malaria endemic region from which it used to be absent until recently. Their short report highlights the public health concern that the invasion of this highly adaptive and efficient malaria vector poses, in this case in a densely populated urban area. This threat has been well recognized and communicated by the World Health Organization (WHO) [1]. Given the presence of ample man-made breeding sites, which *An. stephensi* is well adapted to use to its advantage, the threat of urban malaria outbreaks should not be underestimated; other authors have come to the same conclusions [2, 3]. The authors of the short report also, rightly so, draw attention to the need for an early warning/response system that would quickly detect the introduction of invasive disease vectors before they adapt and establish locally.

We are fully aligned with the authors’ above quoted conclusions but would like to use this opportunity to highlight that their practice of publishing the detection of *An. stephensi* 3 years after identifying the vector in central Khartoum is in rather stark contrast with these. A functioning early warning and response system starts with responsible public health professionals that recognize public health threats and share all information related to them with the relevant public health authorities as quickly as possible. In the present context, the data should have been shared with the Sudanese Federal Ministry of Health and WHO as soon as the vector was morphologically identified. We are concerned that the failure of the authors to follow established reporting procedures could be seen by readers of Parasites & Vectors as legitimate behavior and may therefore set a precedent for others to follow suit. Clearly, late reporting of this nature would be detrimental to WHO’s attempts to coordinate a regional effort across the Horn of Africa to contain the ongoing spread of *An. stephensi*.

In recent years progress in the fight against malaria has stalled. In 2019, malaria caused an estimated 229 million malaria cases in 87 countries and 409,000 deaths [4]. The burden is highest in Africa where it is so far largely, but not exclusively, associated with rural or peri-urban areas, due to the specific habitat preference of the main malaria vectors in the region, *Anopheles gambiae* s.s.,* An. arabiensis*, *An. coluzzii*, and *An funestus*. To date, urban malaria in Africa has been of relatively limited importance. With the invasion of the Horn of Africa by *An. stephensi*, as well as due to rapid urbanization and population growth across the continent, the potential relevance of urban areas for the ongoing global malaria control effort has suddenly changed. Experiences with attempting to control this vector in other settings, such as India, have shown just how difficult a task this can be. Its high level of adaptability, including its readiness to breed in man-made water storage containers that abound in urban areas, have often made it impossible to contain. The recent invasion of the Horn of Africa by *An. stephensi* is just the latest step in the expansion of the vector geographical range and should have put all of us on highest alert. In a context where malaria control has already stalled and financial resources have flat-lined, this recent development provides immense new challenges for the fight against malaria. On the upside, however, the large overlap in urban breeding sites of *An. stephensi* with those of *Aedes aegypti*, a highly efficient vector of dengue, provides a number of opportunities, such as for integrated surveillance and control of the aquatic stages of these two vectors, and by doing so to translate the Global Vector Control Response 2017–2030 into action [5].

In recognition of this new threat to malaria control in Africa, WHO issued a Vector Alert in August 201﻿9 [1], added a dedicated theme on invasive malaria vectors to Malaria Threats Map (https://apps.who.int/malaria/maps/threats), populated this platform with the available data and developed and disseminated a specific data collection form to all Member States and their implementing partners. Efforts to delineate the currently affected geographical area in the Horn of Africa, led by the respective Ministries of Health, the US President’s Malaria Initiative, and national and international research institutions have been stepped up. A revised key to the females of the Afrotropical *Anopheles* mosquitoes that includes *An. stephensi* has been published [6], and been translated into French (https://www.who.int/fr/publications-detail/WHO-UCN-GMP-2021.04) and Arabic to help insure the vector is not mis-identified during entomological survey. Entomological training has been organized by a number of partner institutions to build capacity in this area. In turn, further and more frequent reports of the vector have been received by WHO and been added to the global database, and are being used to informing modelling of the potential areas at risk and the design and implementation of country response plans, and quarterly multi-partner coordination call on *An. stephensi* invasion in Africa are being convened by WHO. While not perfect, the regional response has been initiated and is building up momentum.

A regional response to public health threats such as this one requires partnership whereby all of us put their own interest after those of the common good—saving lives in Africa. The best intentions of national and global health authorities to coordinate a response to *An. stephensi* in the Horn of Africa will remain in vain if data on its presence are traded as academic currency rather than as a public good. Limited resources available for the fight against malaria rely on data to inform rapid targeting of control efforts and, over time, monitoring of the intervention effectiveness. What we see in the short report by *Ahmed* and co-workers is contrary to this internationally galvanized noble spirit that is widely supported by the malaria community, and we feel strongly that any repetition of this practice should be avoided under all circumstance. Our plea to the reader is to share all new data on the presence of *An. stephensi* in the Horn of Africa and from other areas that it may have invaded with the respective Ministry of Health and with WHO. We are ready to receive reports and other enquiries at vectorsurveillance@who.int, we are ready to serve our mandate [7].

## Data Availability

None required.
